# Supplementary material for: Impact of global cooling on Early Cretaceous high pCO2 world during the Weissert Event
Source: Nat Commun. 2021 Sep 13;12:5411. doi: 10.1038/s41467-021-25706-0 (PMC8437947; doi:10.1038/s41467-021-25706-0)
Supplement: Supplementary file 1 — Supplementary Information [file 41467_2021_25706_MOESM1_ESM.pdf]

Impact of global cooling on Early Cretaceous high pCO_2 world during the Weissert Event

SUPPLEMENTARY INFORMATION

1. Chronostratigraphy

The Weissert Event is Late Valanginian in age and Supplementary Fig. 1 illustrates its chronostratigraphy within the Berriasian–Barremian time interval. At low latitudes, integrated nannofossil biostratigraphy, magnetostratigraphy and C-isotopic stratigraphy has been obtained for a number of Tethyan sections, providing the direct intercalibration of events. At higher latitudes, magnetostratigraphy has not been resolved for Lower Cretaceous sections, and chronostratigraphy has been derived from ammonite and calcareous nannofossil biostratigraphy calibrated against chemostratigraphy. It is worth noting that both ammonite and calcareous nannofossil assemblages are, however, affected by marked paleoprovincialism, hampering a direct correlation of bioevents from lower and higher latitudes. As a consequence of such differences in stratigraphic instruments, the only common dating tool is the positive carbon isotopic excursion (CIE) in the $\delta^{13}C$ record associated with the Weissert Event.

Numerical ages of onset and termination - as well as phases within - of the Weissert Event, and stage and sub-stage boundaries depend on the adopted timescale. Currently, there are two groups of available geochronologies constructed using different approaches, namely the geologic timescale of Gradstein et al. (2012)[1] revised by Ogg et al. (2016)[2] and recently updated by Gradstein et al. (2020)[3] , and the timescale of Channell et al. (1995)[4] revisited by Malinverno et al. (2012)[5] (Supplementary Fig. 1). The backbone of both timescales consists of polarity chrons directed dated by calcareous nannofossils. The position of stage (and sub-stage) boundaries has been revised here following subsequent ad/or implemented and/or revised chronostratigraphy including (when available) ammonite biozones. In Fig. 1, we report the position of stage boundaries as documented in the original papers[4,1,5,2, 3]. Previous work[6] documented ammonite and calcareous nannofossil biostratigraphy in a composite section also yielding magnetostratigraphy and chemostratigraphy, and re-calibrated the Valanginian/Hauterivian boundary. Specifically, the base of the *A. radiatus* ammonite zone and, thus, the base of the Hauterivian was found to correspond to the minor carbon isotope anomaly within magnetic chron CM 10N and nannofossil

NC4a subzone. This substantial chronostratigraphic revision was not adopted in[1, 3 and 5] but it was incorporated in the timescale of Ogg et al. (2016)[2].

In this work we adopt the Malinverno et al. (2012)[5] timescale revised according to Ogg et al. (2016)[2] for the position of the Valanginian/Hauterivian boundary relative to polarity chrons (Supplementary Fig. 1).

In the chronostratigraphic scheme (Supplementary Fig. 1) the calcareous nannofossil zonations are reported for the lower[7] and higher latitudes[8]. The stratigraphic position of two calcareous nannofossil events has been revised as follows: i) the FO of *R. wisei*, previously reported before the positive CIE[7], correlates with the ramp of the positive CIE of the Weissert Event as documented in some section in the Vocontian Basin[9,10], in the Central Atlantic[11] and SE Spain[12]; ii) the the last occurrence (LO) of *T. verenae*, previously coinciding with the highest value of the positive Weissert CIE[7], is now placed slightly after the termination of the Weissert Event as reported in several sites[9, 11, 13, 10].

The Boreal zonation takes into account a revision[14] that places the LO of *M. speetonensis* in the topmost part of the positive Weissert CIE.

In the chronostratigraphic scheme we additionally reported those calcareous nannofossil events (in blue), which have been used to stratigraphically constrain the age of Site 692 (Supplementary Figs. 1 and Fig.2).

The Boreal calcareous nannofossil BC4–BC7 zones have been calibrated against the carbon isotopic curve considering the dataset from the Rødryggen section in Greenland[15, 14] , the Wawal section in Poland[16], the BGS 81/43 Site in the North Sea[17] and the revision[14]; while the age of Zones BC1–BC3 and BC7–BC10 is based on the Boreal ammonites inter-calibrated with the Tethyan biochronology as specified in[14].`

2. Calcareous Nannofossils

Calcareous nannofossils were investigated in the Valanginian–Early Hauterivian interval, from 78.61 to 93 mbsf (cores 113–692–10R and 113–692–12R), consisting of a calcium carbonate-rich and overall finely laminated black shale section. A high-resolution sampling at 10 to 20 cm was carried out,

leading to a set of 69 samples. In this work, we also revised the originally studied[18] calcareous nannofossil biostratigraphy of Site 692 that assigned a Valanginian age to the interval from 93 mbsf (113–692–12R CC) to 53.6 mbsf (113–692–7R 1, 44 cm). Calcareous nannofossil assemblages are dominated by high latitude taxa, but cosmopolitan species are also common and a few typically Tethyan species were observed. A biozonation for high latitudes of the southern hemisphere is not available and, therefore, biostratigraphy is assigned with reference to the standard zonation scheme for the Boreal Realm (BC[8] zonation) and the Tethys (NC[7] zonation). Previous nannofossil investigations of Site 692[18] adopted the NC[19] zonation.

At date, there is no available intercalibration of low and high latitude nannofossil biozonations. However, the direct correlation of a few nannofossil biohorizons relative to chemostratigraphy exists for sections in a variety of basins (e.g.,[20,21,6,9,11,15,22,10,14,12]) allowing indirect tie-points. Moreover, following the lower[7] and higher[8] latitude zonations, revised age assignments were documented for a few nannofossil events and such updates were incorporated in the chronostratigraphic framework applied in this work (Supplementary Fig. 1).

The majority of samples investigated at Site 692 contain relatively diverse and moderate to well preserved nannofossils although the assemblages are dominated by few species, most prominently *Watznaueria barnesiae* and, in the interval comprised between 91.87 mbsf (113–692–12R–3, 65–68 cm) and 80.52 mbsf (113–692–10R–2, 50–54 cm), by *Biscutum constans* and *Crucibiscutum salebrosum*. Spare samples in core 113–692–12R (91.31, 90.57, 90.42, 89.4, 89.22, 88.93, 88.78 and 88.50 mbsf) are barren of nannofossils. The lowermost studied sample (sample 113–692–12R–3, 112–115 cm) is characterized by the presence of the Boreal taxon *Crucibiscutum ryazanicum* and the Tethyan taxon *Percivalia fenestrata*, which indicate an age not older than late Berriasian, within Zone BC2[8] and subzone NC2b[7] (Supplementary Fig. 2).

The absence of Boreal *Triquetrorhabdulus shetlandensis* and Tethyan *Calcicalathina oblongata* marker species for the base of the Valanginian does not allow a more precise age determination of the interval between 93 and 91.87 mbsf (sample 113–692–12R–3, 65–68 cm) that is attributed to the Late Berriasian–Early Valanginian BC2–BC3 and NC2b Zone (Supplementary Fig. 2).

The first occurrence (FO) of *Zeugrhabdotus trivectis* was observed at 91.87 mbsf, shortly before the onset of the positive $\delta^{13}C$ isotopic CIE of the Weissert Event (Supplementary Fig. 2). This biohorizon has been documented in the lowermost part of Zone NC3 in sections from the Vocontian Basin[9, 22, 10], SE Spain[12] and DSDP Site 535 in the Gulf of Mexico[17] (Supplementary Fig. 1). According to previous work[8], the FO of *Z. trivectis* falls within zone BC4.

In well-dated sections from the Vocontian Basin (France) and Betic Cordillera (Spain), the LO of *Eiffellithus primus* was found above the FO of *Z. trivectis,* and below the FO of *R. wisei* at the base of the $\delta^{13}C$ isotopic positive CIE[9, 10, 12]. At Site 692 the position of the LO of *E. primus* is comparable (sample 113–692–12R–2, 105–110 cm; 90.78 mbsf) (Supplementary Fig. 2).

The LO of the Tethyan species *Rucinolithus wisei* at 89.02 mbsf (sample 113–692–12R–1, 80–83 cm) marks the base of subzone NC3b[7] and is Late Valanginian in age as documented in several low latitudinal sites[9, 11, 22, 10, 12] (Supplementary Fig. 1). At Site 692, the LO of *R. wisei* corresponds to the early phase of the $\delta^{13}C$ positive CIE (Supplementary Fig. 2).

At Site 692, disarticulated elements of possible *Micrantolithus speetonensis* occur in the interval comprised between 84.03 mbsf (sample 113–692–10R–4, 101–104 cm) and 83.74 mbsf (sample 113–692 –10R–4, 73–75 cm) in correspondence of the topmost part of the $\delta^{13}C$ isotopic positive CIE (Supplementary Fig. 2). The LO of *M. speetonenesis,* marking the base of Zone BC5[8], was originally identified as latest Early Valanginian in age[8, 23], but a recent revision[14] places this event in the Late Valanginian, close to the topmost part of the positive carbon isotope excursion of the Weissert Event (Supplementary Fig. 1). The stratigraphic position of disarticulated *M. speetonensis* at Site 692 is, thus, consistent with the revision[14].

At Site 692, the boundary between zones NC3 and NC4 cannot be determined because *Tubodiscus verenae* is absent. However, the LO of *T. verenae* is calibrated with respect to the chemo- and magnetostratigraphy and correlates with the topmost part of magnetochron CM11 and just above the Weissert Event CIE. Consequently, at Site 692 we tentatively place the NC3/NC4 zonal boundary between 82 mbsf and 83 mbsf (Supplementary Fig. 2).

At Site 692, the position of the Valanginian/Hauterivian boundary based on calcareous nannofossils is not straightforward due to the absence of low and high latitude markers such as *Tubodiscus verenae* [7] and *T. shetlandensis* and/or *Eprolithus antiquus* [8], respectively. However, the Valanginian/Hauterivian boundary may be constrained using the last common occurrence (LCO) of *C. deflandrei* detected at 80.98 mbsf (sample 113–692–10R–2, 96–99 cm) (Supplementary Fig. 2). In fact, in Tethyan and low latitude sections, the LCO of *C. deflandrei* was documented in the latest Valanginian after the LO of *T. verenae* within subzone NC4a and close to the Valanginian/Hauterivian boundary[24, 19, 25.] With respect to the carbon isotopic curve ($\delta^{13}C$), the LCO of *C. deflandrei* is reported just above the Weissert Event CIE[25] with respect to the $\delta^{13}C$ record of[20]; Roth (1983)[19] with respect to the $\delta^{13}C$ curve of Littler et al. (2011)[26] and Applegate & Bergen (1988)[27] with respect to the $\delta^{13}C$ curve of Kessels et al. (2006)[17]. At Site 692, the LCO of *C. deflandrei* shortly precedes the minor carbon isotope anomaly that was equated to the base of the *Acanthodiscus radiatus* ammonite zone and, thus, to the base of the Hauterivian[6], confirming the potentiality of this nannofossil event to place the Valanginian/Hauterivian boundary.

2.1 Paleoecology of braarudosphaerids

A braarudosphaerid increase in abundance is observed at Site 692 in the relative warming phase following the cooling at the end of the Weissert Event and in the warmer interval following the cold phase across the Valanginian/Hauterivian boundary (Supplementary Fig. 3). Coeval braarudosphaerid-enrichments are documented offshore Antarctica, at ODP Sites 766 and 765[28, 29], and at high latitudes in the northern hemisphere, in the BGS 81/43[17] and Rødryggen section in Greenland[15, 14], where braarudosphaerids are mainly represented by *Micrantholithus hoschulzii* and *Micrantholithus obtusus*. Conversely, latest Valanginian braarudosphaerid blooms have not been recorded at low latitudes in Tethyan sections, where discrete *Micrantholithus* enrichments are observed in upper lower Valanginian[30, 31], upper Hauterivian[32,31] and lower Aptian[31] intervals. In the Vocontian Basin (Angels section[33]) braarudosphaerids (mostly *M. hoschulzii* and *M. obtusus*) are frequent before, during and after the Weissert Event. In Romania[34], in the Galicia Margin (ODP Site 638[27, 17]) and in the Proto North Atlantic

(DSDP Site 603[35]) braarudosphaerids (*M. hoschulzii* and *M. obtusus*) enrichments are detected only after the Weissert Event. Modern braarudosphaerids are often most abundant in low-salinity waters[36, 37] and fossil enrichments of *Braarudosphaera* have been referred to coastal low-salinity waters[38, 39, 30]. The specific occurrence of braarudosphaerid enrichments at high latitudes during the warmer interlude separating the two cold periods and locally shortly after the Valanginian/Hauterivian boundary, suggests seasonal salinity lowering possibly triggered by discharges of fresh deglacial melt water, supporting the possibility of ice in coastal regions.

3. Benthic foraminifera

Benthic foraminifera were studied in 31 samples from Site 692 (from 113−692−10R−1, 25−27 cm to 113−692−12R−3, 112−115 cm) to reconstruct the paleowater depth during the Valanginian−Hauterivian. The most abundant genus recorded at Site 692 is *Eoguttulina*, indicative of a shallow shelf (e.g.,[40,41]) or upper bathyal setting (e.g.,[42]). Less abundant taxa such as *Laevidentalina, Lenticulina, Lagena*, *Saracenaria*, and *Nodosaria* are characteristic for a wide paleobathymetric range, from inner neritic to lower bathyal environments (e.g.,[42, 43]). The genus *Vaginulinopsis* is reported as a bathyal indicator in the Indian Ocean (e.g.,[44]). Based on the benthic foraminiferal assemblage, an outer neritic-upper bathyal (~200–500 m) paleodepth is suggested for the Valanginian–Hauterivian interval at Site 692. A published paleobathymetric division[45, 46] was applied.

4. Anomalous GDGT distributions

The the fraction of crenarchaeol regio-isomer to total crenarchaeol, $f_{Cren':Cren' + Cren}$ (= [Cren']/[Cren] + [Cren'];[47]) is always <0.25 in all study samples, however we note an anomalous isoprenoid-Glycerol Dialkyl Glycerol Tetraether (i-GDGT) distribution in samples from the restricted Early Cretaceous proto-Weddell Sea and proto-North Atlantic. Supplementary Fig. 4 shows a cross-plot of $f_{Cren':Cren' + Cren}$ against TEX_{86} that documents a systematic increase in the relative abundance of crenarchaeol regio-isomer [Cren'] compared to crenarchaeol [Cren] in the Early Cretaceous Atlantic Ocean (red dots in Supplementary Fig. 4;[47]) and in the Weddell Sea (Site 692; green rectangles in

Supplementary Fig. 4; this study and 12 other Early Cretaceous data from this site[26]). Therefore, the Early Cretaceous Atlantic Ocean and Weddell Sea samples, similarly to the present-day deep restricted basins of the Mediterranean and Red Sea (yellow rectangles in Supplementary Fig. 4;[48,49]), document a considerable deviation from values observed in the modern core-top dataset (empty grey circles in Supplementary Fig. 4;[50]) and the rest of Cretaceous data (black dots in Supplementary Fig. 4;[47]). Moreover, we also document high [GDG-2]/[GDGT-3] ratios at Site 692 that range between 4 and 8.5, similar to deeper sediments from the modern Mediterranean Sea (see[49 and 51]). To date, a possible explanation for these distinct sedimentary i-GDGT distribution documented in the proto-North and South Atlantic[51] and Southern Ocean Basin (proto- Weddell Sea [this study]) might be peculiar of young and restricted Mesozoic ocean basins, leading to a different TEX_{86}-temperature relation[51], as documented in the modern Mediterranean and Red Sea[49].

It is important to note that the paleoceanographic setting and resulting circulation of the Valanginian Weddell Sea are different from the present-day Eastern Mediterranean and Red Sea. The paleolatitude of ~54 °S is consistent with an overall more humid climate based on modern day climate zonation. While this is clearly different to the modern evaporative Mediterranean Sea, we argue that the increased surface freshwater input combined with deoxygenated bottom waters is a better analogue to times of sapropel formation in the Mediterranean Sea. Importantly, a downcore study[52] showed that regional TEX_{86}-SSTs are consistently warmer (up to 15 °C) than U^{K}_{37}-derived temperatures, both inside and outside of Pleistocene sapropels. This is clear evidence that the occurrence of endemic Thaumarchaeota populations in the restricted basin can change the local TEX_{86}-SST relation independent of large swings in water column stratification and oxygenation. We therefore argue that the young and restricted basins of the Early Cretaceous, in analogue to the modern Mediterranean and Red Sea, might also provide special environmental conditions enabling specific Thaumarchaeota community structures and potentially different TEX_{86} export dynamics than commonly considered in the present-day global core-top calibrations.

5. Organic matter quality and thermal maturation

Carbon isotope and TEX_{86} analysis was supplemented by analysis of thermal maturity characteristics by Rock-Eval pyrolysis and GC–MS analysis of the aliphatic hydrocarbon fraction in order to determine the quality of the organic matter in the studied stratigraphic interval (cores 113–692–10R and 113–692–12R) of Site 692. The aliphatic fraction (purified as described above) was performed on a Thermo Scientific Trace 1310 GC fitted with a split splitless injector (at 280 °C) and linked to a Single Quadrupole Mass Spectrometer using electron ionization (electron voltage 70eV, source temperature 230 °C, quadrupole temperature 150 °C, multiplier voltage 1800V, interface temperature 310 °C). Data acquisition and processing was carried out using Thermo Chromeleon 7 software. Samples were analysed in full scan acquisition mode (50–600 amu/sec) and in selected ion monitoring (SIM) mode. Samples were diluted in hexane and injected onto a Thermo fused silica capillary column (60 m x 0.25 mm i.d) coated with 0.25 μm 5 % phenylmethylpolysiloxane phase using an auto sampler. The GC temperature was increased from 50 to 310 °C at a rate of 5 °C/min. The final temperature was held for 10 minutes. Helium was used as the carrier gas (flow rate of 1 ml/min, initial inlet pressure of 50 kPa, split at 30 ml/min).

Our samples yielded consistently high $\beta\beta/(\beta\beta+\alpha\beta+\beta\alpha)$-$C_{31}$-homohopane ratios of ~0.5–0.6 indicating the presence of immature organic matter[53], consistent with previous work[26] on thermal maturity assessment of Early Cretaceous samples from Site 692.

Rock-Eval pyrolysis was performed on aliquots of ~100 mg of powdered core samples, with each sample analysed in duplicate and a standard sample analysed after every 10 samples. The samples were heated at 100 °C for three minutes, during which free volatile hydrocarbons (gas) were released (S_0). The temperature was then increased from 100 °C to 300 °C and held for three minutes to allow the expulsion of free hydrocarbons (oil) from the sample (S1). The furnace temperature was then ramped from 300 °C to 550 °C at a rate of 25 °C /minutes and held at 550 °C for 2 minutes; the S2 peak and the T_{max} (°C) were recorded at this stage.

Results indicates mainly Type II/III kerogen (HI ~400, OI ~65) with low thermal maturity (T_{max} values ~420 °C). These additional data are consistent with published data from Site 692[18] and further support a low thermal maturity, which had a negligible impact on TEX_{86} values.

6. Global mean surface temperature and pCO_2 estimates

We compare the reconstructed Valanginian ocean temperatures with the simulated sea surface temperatures (SSTs) at the individual paleopositions (see Supplementary Table 1) to calculate global mean surface temperature (GMST) estimates for each site. We follow the approach described in[54] that assumes a linear relation between changes in local and global mean surface temperatures. We further assume that our model can capture this relation and we can therefore use our ×2 and ×4 CO_2 simulations to estimate the GMST ($<T>^{inferred}$) from a local proxy temperature (T^{proxy}) as

$$< T >^{inferred} = < T^{x2} > + (T^{proxy} - T^{x2}) \frac{<T^{x4}> - <T^{x2}>}{T^{x4} - T^{x2}} \qquad (3)$$

where $<T^{x2}>$ and $<T^{x4}>$ are the GMST of the ×2 and ×4 CO_2 simulations, respectively, and T^{x2} and T^{x4} are the local model SSTs from the same simulations. T^{x2} and T^{x4} are averaged over a horizontal area of ±1 grid point (±2.5° latitude and +/-3.8 ° longitude) around the rotated paleolocations to account for paleogeographic uncertainties. $<T>^{x2}$ and $<T>^{x4}$ for the Valanginian are 17.28 °C and 21.03 °C, respectively. We note that any GMST estimate for a proxy temperature smaller than T^{x2} or larger than T^{x4} is based on extrapolated instead of interpolated model temperatures and is therefore more uncertain. Illustrations of the results of this method for all sites are shown in Supplementary Fig. 5. Proxy temperatures for locations 10 and 19 from Table S1 result in extreme GMST estimates above 30 °C and below 10 °C, respectively (Supplementary Figs. 5j and p). We note that the reconstructed temperatures based on oxygen isotopes at location 10 might be influenced by diagenetic alterations (see discussion below), while the authors of the original publication of location 19[55] argue that the absolute Mg/Ca-derived temperatures are highly uncertain. We therefore exclude GMST estimates from locations 10 and 19 from the calculation of the average GMSTs. Exclusion of both sites results in virtually identical mean GMST estimates, but with a reduced standard error of the mean. We still include the relative temperature changes towards the Weissert CIE end at both sites (Supplementary Table 1) to calculate the root-mean-square-errors between simulated and reconstructed surface cooling (Figure 4c of the article, Supplementary Figs. 6e and f). Fig. 3 in main text indicates that most

available records show relatively stable temperatures across the Early-Late Valanginian transition and virtually no warming associated with the onset of the Weissert CIE. One contrasting observation suggesting significant warming in the proto-North Atlantic and Tethys realm based on $\delta^{18}O_{carb}$-based temperature reconstructions[56]. We note that this reconstruction is in stark contrast with other temperature proxies from same locations (e.g., TEX_{86};[26]; $\delta^{18}O$ of belemnites[57, 58]; Mg/Ca ratios[55]), and therefore conclude that the causes for these marked discrepancies are likely related to a diagenetic alteration of the $\delta^{18}O_{carb}$ values, which were measured on bulk rock samples. We therefore focus our model-data comparison on only two distinct temperature periods. The Weissert warm interval includes Valanginian average temperature estimates older than the first Weissert CIE peak (red bars below point B in Fig. 3 in the article). The cold Weissert end includes the coldest temperature reached at the end of the Weissert CIE (dark blue color interval coincident with point C in Fig. 3 in the article).

We calculate mean GMST estimates $< T >_{avg}^{inferred}$ as the average over all proxy locations for each period and derive uncertainty estimates as the respective standard error of the mean. We can then calculate a global mean scaling factor s by

$$s = \frac{<T>_{avg}^{inferred} - <T^{x2}>}{<T^{x4}> - <T^{x2}>} \qquad (4)$$

to linearly scale simulated surface temperatures to the estimated $< T >_{avg}^{inferred}$ by

$$T^{scaled} = T^{x2} + s * (T^{x4} - T^{x2}) \qquad (5)$$

Temperature distributions for T^{scaled} are shown in Figs. 4 and 5 in the article as well as in Supplementary Fig. 6. We further use T^{scaled} to calculate the root-mean-square-error (RMSE) between best-fit model temperatures and local proxy data. The same scaling factor can also be used to derive the associated best-fit model CO_2 concentration by

$$CO_2^{inferred} = 560ppm * \left(\frac{1120ppm}{560ppm}\right)^s \qquad (6)$$

The shown temperature changes towards the Weissert CIE end (cooling) follow as the difference between T^{scaled} of both time intervals with uncertainties derived from the error propagation of both time periods. We perform the GMST estimation both using annual mean model temperatures at all

sites (annual mean assumption) as well as using warmest 3-month means for mid to high-latitude (>45 °) sites (summer bias assumption) to assess the influence of a potential seasonal bias in the proxy signal formation (see discussion in the article).

We perform the analysis with five different variations of the proxy data to test how individual proxy techniques and calibration choices influence the resulting GMST and pCO_2 estimates. First, we include all data for each time period but apply either the calibration for modern deep, restricted basins[49] (min-TEX_{86}, Equation 2 in Methods section in the article) or the BAYSPAR calibration[59, 50] (max-TEX_{86}) to DSDP/ODP sites 534, 603 and 692. The higher T^{proxy} from the BAYSPAR calibration (Supplementary Fig. 6) in turn leads to higher GMST estimates $<T>^{inferred}$ (Supplementary Equation 4) for these sites than in min-TEX_{86} and the overall average GMST for both time periods increases by 4–6 °C (Supplementary Table 2). Associated model pCO_2 concentrations for the Weissert warm interval would be above 2500 ppm - even for the summer bias assumption (Supplementary Fig. 6) - and therefore higher than the ~1700 ppm reported as the upper 95% confidence limit of a multi-proxy CO_2 compilation[60]. Furthermore, the scaled temperature distributions in the model at these high GMSTs show higher RMSE scores (e.g., worse model-data fit) than in min-TEX_{86} (Supplementary Fig. 6). We further perform three different subsampling experiments where we only include estimates from single proxy techniques (only-TEX_{86}, only-$\delta^{18}O$ and only-Δ47) to test whether our results are consistent across the different proxy archives. Note that subsampling experiment only-Δ47 has only been performed for the Weissert warm interval due to the lack of Δ47-derived temperatures for the Weissert cold CIE end. All three subsampling experiments show similar results for the Weissert warm interval with GMST estimates well within reported uncertainties of the mean (Supplementary Fig. 6). Sensitivity to the proxy method is larger for the Weissert cold end with higher GMST estimates derived from TEX_{86} than $\delta^{18}O$ samples. The Experiment only-TEX_{86} includes the respective minimum TEX_{86} temperatures and shows overall highest consistency to the scaled model temperatures (e.g., lowest RMSE), while we find largest uncertainties in the comparison with the oxygen isotope record (experiment only-$\delta^{18}O$) of both time intervals.

FIGURES AND TABLES

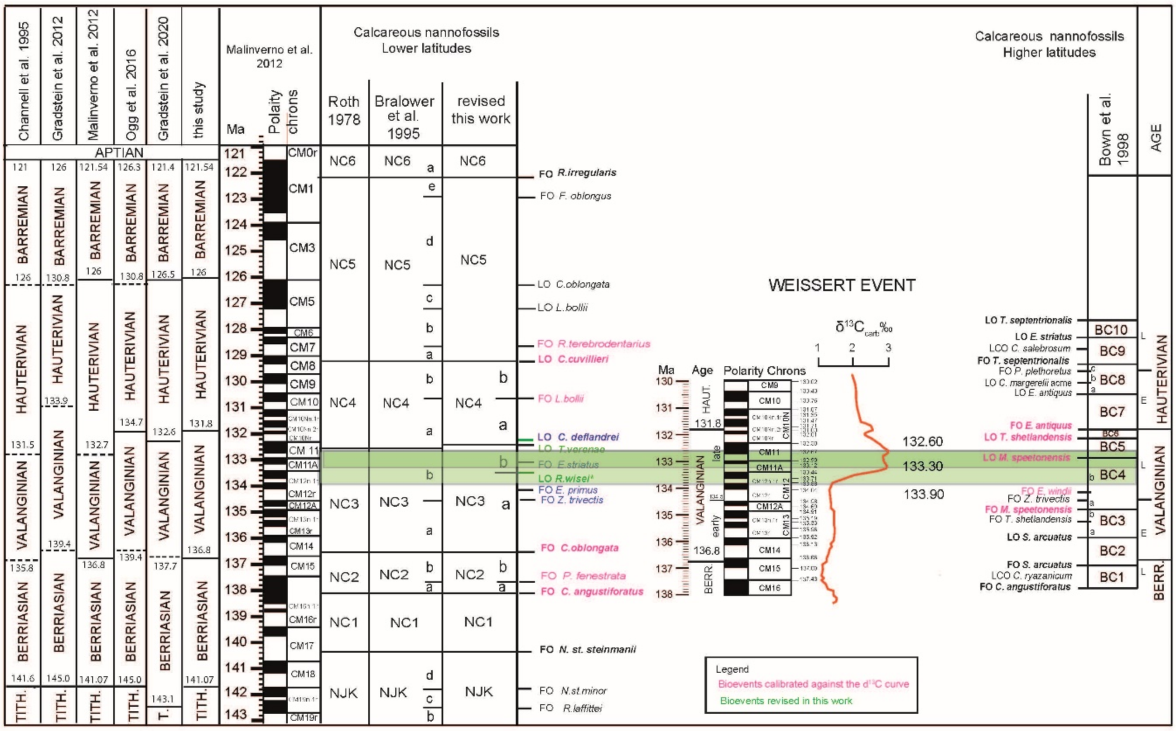

Supplementary Figure 1. **Revised chronostratigraphy and ages of the Berriasian–Barremian interval (this study).** Detailed revised nannofossil biostratigraphy integrated, magneto- and chemostratigraphy across the Valanginian–Hauterivian time interval are also provided. Numerical ages of the Weissert ($\delta^{13}C_{carb}$[6]) are based on the timescale[5] revisited in this study.

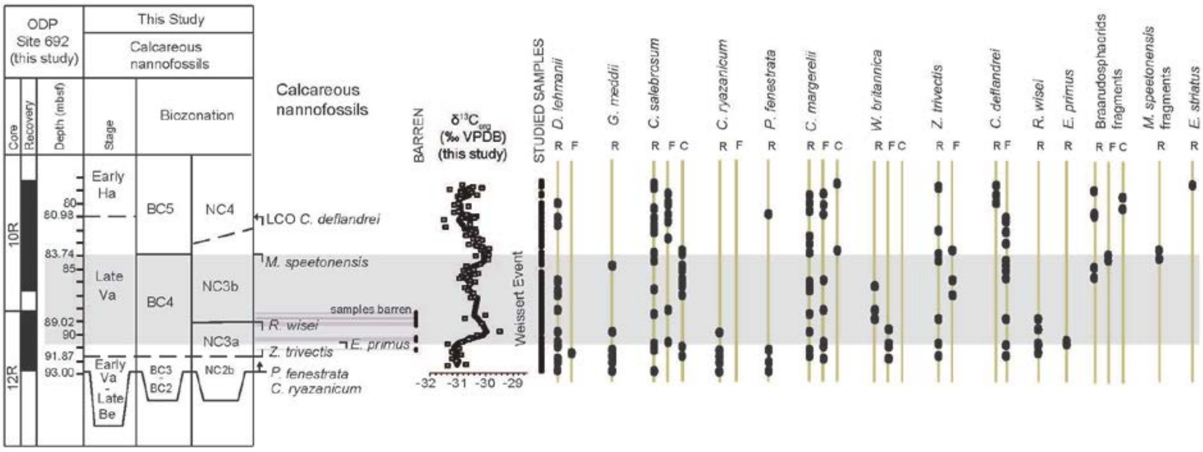

Supplementary Figure 2. **Calcareous nannofossil biostratigraphy of ODP Site 692 (this study).** Calcareous nannofossil zonations include the Boreal Realm (BC[8] zonation) and the Tethys (NC[7] zonation). The relative abundance of nannofossil species relevant for biostratigraphy are reported. R=Rare, 1 specimen in >30 fields of view; F=Frequent, 1 specimen in 11–30 fields of view; C=Common, 1 specimen in 2–10 fields of view. LCO=Last Common Occurrence. The dark grey lines correspond to samples barren of nannofossils. The carbon isotopic curve is from this work. The grey band corresponds to the Weissert Event interval.

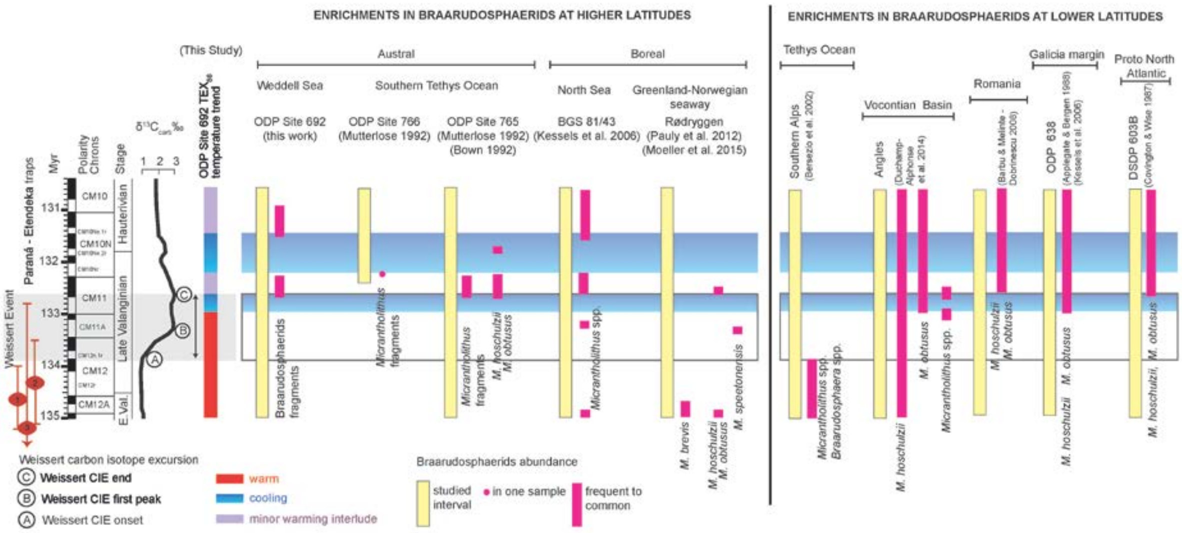

Supplementary Figure 3. **Intervals of braarudosphaerid enrichments at higher and lower latitudinal sites across the Weissert Event interval (this study).** Numerical ages are based on the timescale[5] revised in this work. Identification of the Weissert positive carbon isotope excursion (CIE) is based on the carbon isotope record measured from bulk carbonate ($\delta^{13}C_{carb}$)[6], which has been calibrated in the Southern Alps in the uppermost part of magnetic chron CM12 (A, CIE onset) and in the upper part of magnetic chron CM11 (C–CIE end) [21]. Temperature intervals include the literature data and the new paleotemperatures reconstructed in the current work as discussed in the article (see Fig.3 in the article). Paraná–Etendeka timing after (1)[61], (2)[62] and (3)[63].

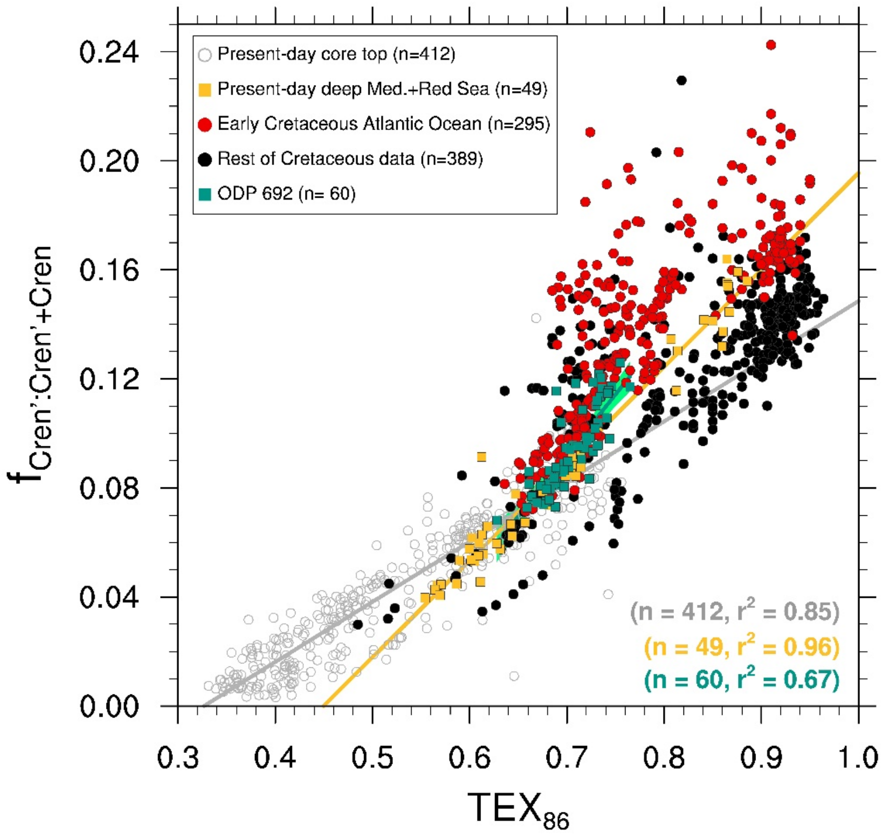

Supplementary Figure 4. **Cross-plot of fractional abundances of crenarchaeol regio-isomer to total crenarchaeol ($f_{Cren':Cren' + Cren}$) against TEX_{86}.** Data show a modern global dataset of marine core-tops and surface sediments (empty grey circles;[50]); present-day samples from deep (below 1000 m) restricted basins of the Mediterranean and Red Sea (yellow rectangles;[48, 49]); a Cretaceous compilation (black dots;[47]) that also includes Early Cretaceous Atlantic Ocean samples (red dots;[47]); and the Early Cretaceous Weddell Sea dataset (ODP Site 692; green rectangles including new Valanginian data from this study and 12 other Early Cretaceous data from this site[26]). All samples with ring index |ΔRI| >0.3 and present-day samples with sea surface temperatures (SST_s) <5 °C are excluded from this analysis for comparability to previous work[51].

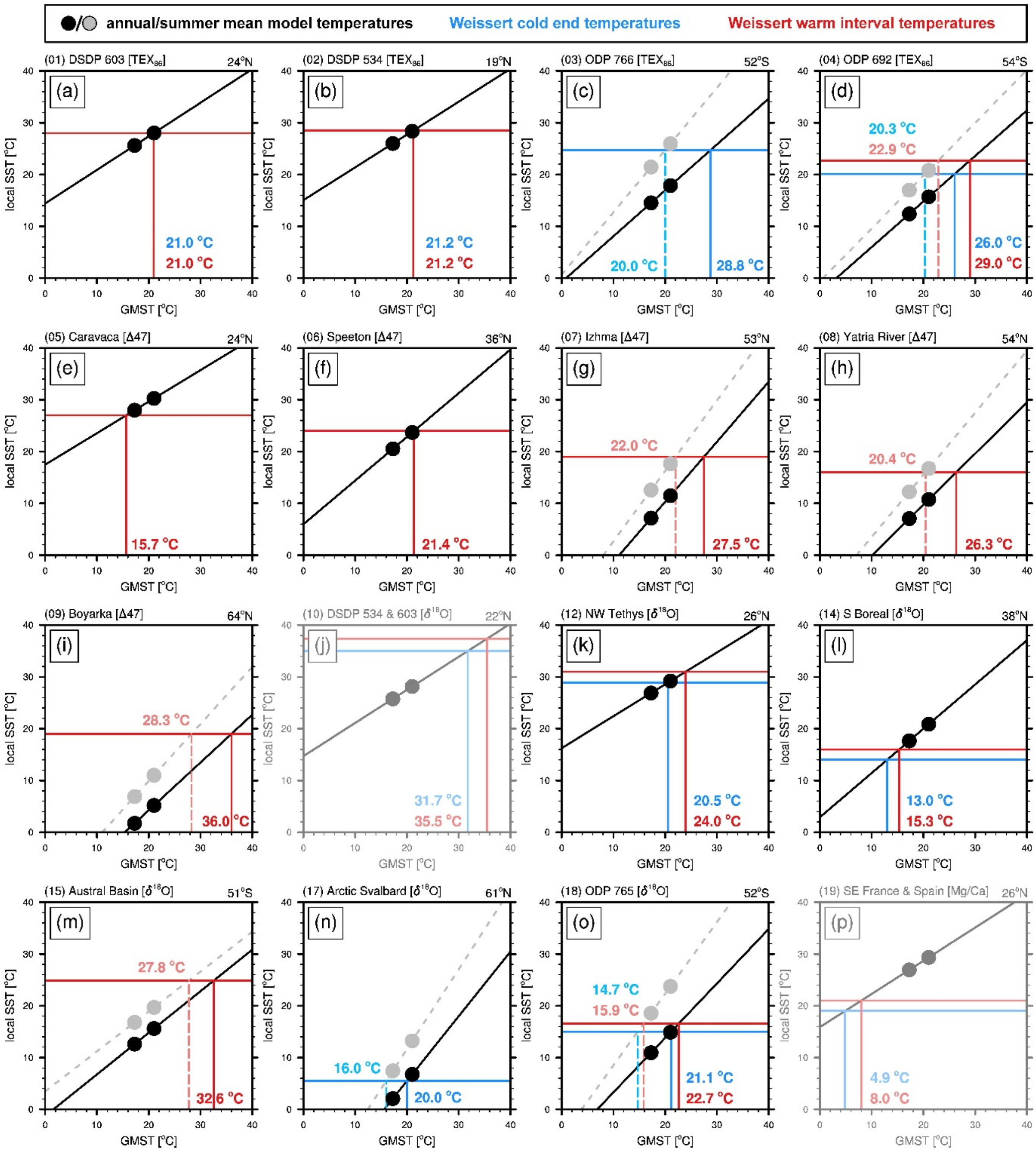

Supplementary Figure 5. **Illustration of global mean surface temperatures (GMST) estimates for all sites (Supplementary Table 1) calculated with Supplementary Equation 3**. **a – p** Results from experiments from single proxy techniques (only-TEX_{86} (a-d), only-Δ47 (e-i), only-$\delta^{18}O$ (j-o), only-Mg/Ca (p). The vertical line shows $\langle T\rangle^{\mathrm{inferred}}$ for annual mean (bold line) and 3-month summer mean temperatures (stippled line) and the horizontal line shows T^{proxy} for sites covering the (red) Weissert warm interval and (blue) Weissert cold carbon isotope excursion end. The black dots show $\langle T^{2x}\rangle$ and T^{2x} as well as $\langle T^{4x}\rangle$ and T^{4x} derived from the model (where $\langle T^{x2}\rangle$ and $\langle T^{x4}\rangle$ are the GMST of the ×2

and ×4 CO_2 simulations). Calculations are repeated with 3-month summer mean temperatures for sites poleward of 45 ° (grey circles) that lead to overall lower GMST estimates. Linear regression lines are presented for annual mean (black) and 3-month summer mean temperatures (grey). Proxy temperatures for sites in shaded panels (j) and (p) result in extreme GMST estimates above 30 °C and below 10 °C and are excluded from the subsequent mean GMST estimates.

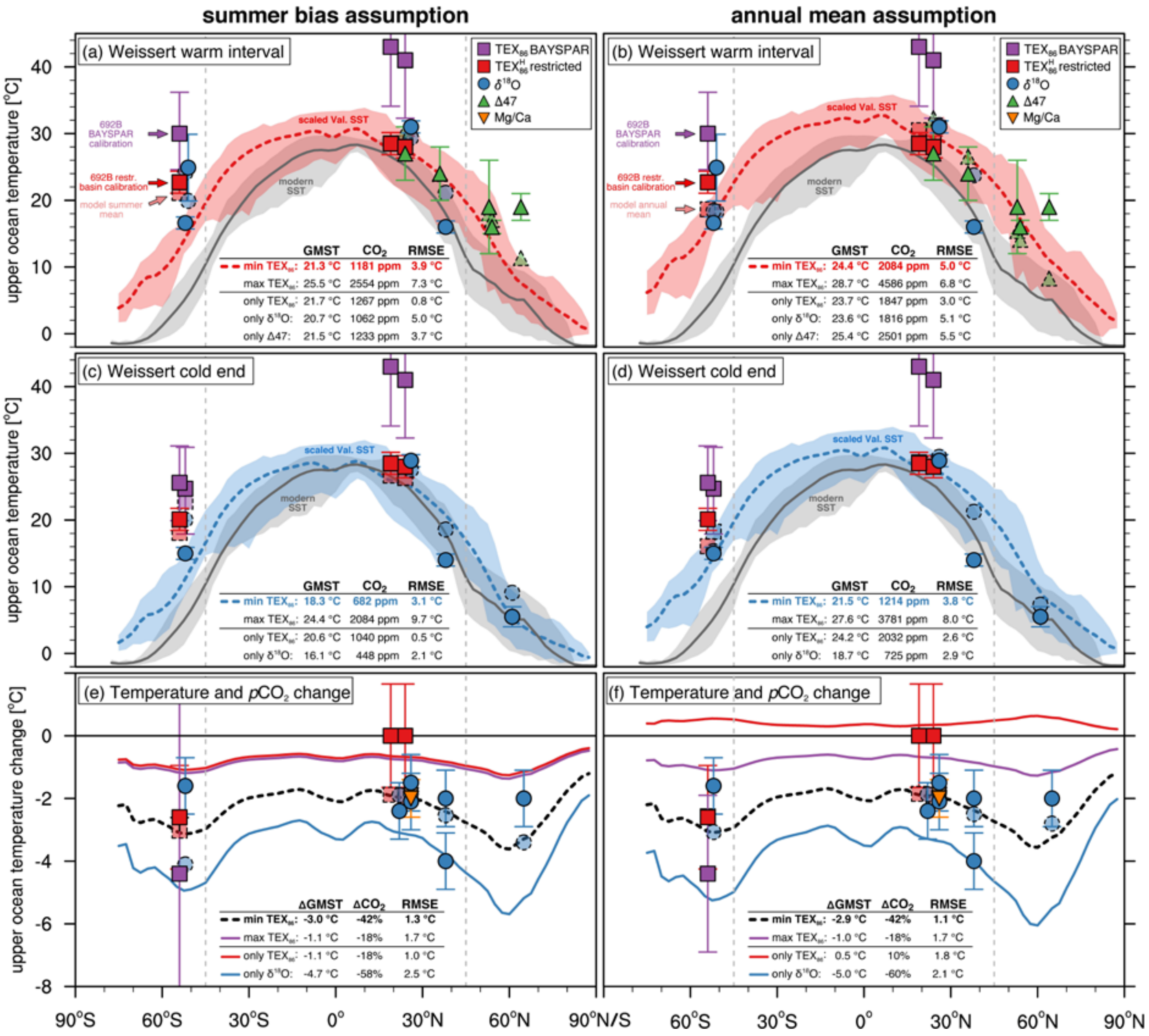

Supplementary Figure 6. **Model-proxy comparison of upper ocean temperatures and model CO_2 estimates.** The model-data comparison is performed both with 3-month summer mean temperatures for sites poleward of 45° (summer bias assumption; first column) as well as annual mean model data (annual mean assumption; second column). The results for each calculation are presented as a table in the respective sub-figure. Global mean surface temperatures (GMSTs) and associated model CO_2 levels are calculated for the whole data set and repeated for three different subsampling experiments including only single proxy techniques (only-TEX_{86}, only-$\delta^{18}O$ and only-$\Delta 47$). Moreover, either the calibration for modern deep, restricted basins (min-TEX_{86}) or the BAYSPAR calibration (max-TEX_{86}) for DSDP/ODP sites 534, 603 and 692 are included. Model sea surface temperatures (SSTs) are linearly scaled to the respective mean CO_2 estimates (Supplementary Equation 5) with root-mean-square-error (RMSE) between scaled model temperatures and local proxy data. Full symbols represent

direct temperature reconstruction from proxy-data with error bars documenting 90% confidence intervals for the TEX_{86} calibration and uncertainties for other proxies as reported in Supplementary Table 1. Dashed symbols represent model SSTs at the proxy locations. Shading around the zonal mean shows the range of annual mean SSTs at each latitude, for modern (grey) and Valanginian (red for Weissert warm and blue for Weissert cold end). Modern SST range (HadISST,[64]) is averaged for the period 1990–2019. **a – b** Weissert warm interval with CO_2 estimates of 1181 ppm. **c – d** Weissert cold end with CO_2 estimates of 682 ppm. **e – f** Model temperature and pCO_2 change from the Weissert warm interval to Weissert cold end.

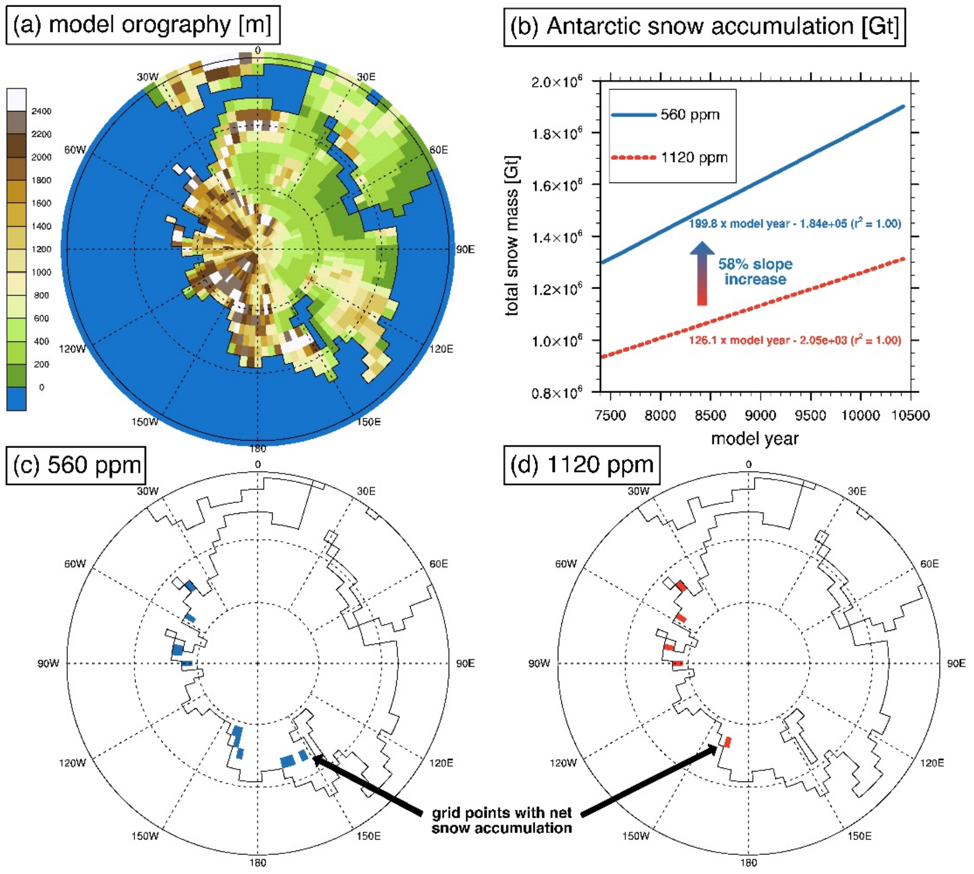

Supplementary Figure 7. **Atmospheric CO_2 sensitivity of simulated Antarctic snow accumulation.** **a** Model orography used for both simulations. **b** Integrated February snow mass poleward of 60 °S for the last 3000 model years. Absolute Antarctic snow masses increase throughout the simulations indicating a net freshwater transport onto Antarctica at both CO_2 levels. Calculated slopes show an increase of 58% in annual mass accumulation rates (from 126 to 200 Gt/year) for an atmospheric CO_2 reduction from ×4 to ×2 pre-industrial levels. **c – d** Panels show regions with a positive mass balance where the snow accumulates.

Proxy	ODP DSDP Sites/ global sections	Location	Location in Fig.1 in the article	Model Latitude	Weissert warm interval [°C]	Weissert cold end [°C]	Cooling (ΔT)	Temp. calib.	Weissert carbon isotope excursion ($\delta^{13}C$)	Ref.	Notes
TEX_{86}	DSDP 603	Proto North Atlantic	1	24°N	~41/ 28±1°C	~41/ 28±1°C	0	max/ min TEX_{86} calib.	✓	[26,47]	
TEX_{86}	DSDP 534	Proto North Atlantic	2	19°N	~43/ 28.5±1°C	~43/ 28.5±1°C	0	max/ min TEX_{86} calib.	✓	[26,47]	
TEX_{86}	ODP 766	Exmouth Plateau	3	52°S	-	-	~25.5	max TEX_{86} calib.	✗	[26,47]	25.5=avg. temperatu-re calculated from the 8 lowermost data points at Site 766 that sits slightly above the Weissert CIE end.
TEX_{86}	ODP 692	Proto Weddell Sea	4	54°S	~30/ 22.7±1°C	~25.6/ 20.1±1°C	~3/4	max/ min TEX_{86} calib.	✓	[this study]	
Δ47	Caravaca	Spain	5	24°N	~27 (±4)	-	-	calcite calib. [65]	✗	[66]	
Δ47	Speeton	Southern Boreal	6	36°N	~24 (±4)	-	-	calcite calib. [65]	✗	[66]	
Δ47	Izhma	Arctic Boreal	7	53°N	~19 (±7)	-	-	calcite calib. [65]	✗	[66]	
Δ47	Yatria River	Arctic Boreal	8	54°N	~16 (±1)	-	-	calcite calib. [65]	✗	[67; 66]	
Δ47	Boyarka	Arctic Boreal	9	64°N	~19 (±2)	-	-	calcite calib. [65]	✗	[66]	
$\delta^{18}O$ bulk carbonate	DSDP 603 and 534	Proto North Atlantic	10	22°N	~37.4 (±0.9)	~35	~2.4	calib. [68] using 0.5‰ $\delta^{18}O$ seawater	✓	[56]	37.4=avg. values from Late Val. transition to Late Val. interval in[13]. Trends may be bias due to large scattering and/or diagenetic overprint of the bulk carbonate signal.

$\delta^{18}O$ belemnite	Compila-tion	South East France	11	26°N	-	-	~1-2	-	✓	[57; 58]	Authors suggest that the positive shift in $\delta^{18}O$ is equivalent to a ~1-2°C cooling but absolute tempera-tures are highly uncertain.
$\delta^{18}O$ bulk carbonate	Compila-tion	North West Tethys	12	26°N	~31.0 (±0.9)	~28.9 (±0.9)	~2.1	calib. [68]; avg. for 1‰ and 1.4‰ $\delta^{18}O$ seawater	✓	[56]	31.0=avg. values from Late Val. transition to Late Val. interval in[13]. Trends may be bias due to large scattering and/or diagenetic overprint of the bulk carbonate signal.
$\delta^{18}O$ belemnite	Compila-tion	Southern. Boreal	13	38°N	-	-	~4	-	not clear	[58]	Authors suggest that the positive shift in $\delta^{18}O$ is equivalent to a ~4°C cooling but absolute tempera-tures are highly uncertain.
$\delta^{18}O$ lenticuli-na +bivalve	Compila-tion	Southern Boreal	14	38°N	~16.0 (±0.9)	~14 (±0.9)	~2	calib.[68]; avg. for 0‰ and −0.5‰ $\delta^{18}O$ seawater	not clear	[56]	
$\delta^{18}O$ belemnite	Rio Guanaco	Austral Basin	15	51°S	~24.9 (19-30)	-	-	calib.[68] using -1.2 ‰ $\delta^{18}O$ seawater	not clear	[69]	

$\delta^{18}O$ belemnite	Compilation	Arctic Boreal	16	65°N	-	-	~2	-	not clear	[58]	Authors suggest that the positive shift in $\delta^{18}O$ is equivalent to a ~2°C cooling but absolute temperatures are highly uncertain.
$\delta^{18}O$ belemnite co-occurring with glendonites	Festningen and Janusfjellet	Arctic Svalbard	17	61°N	-	~5.5 (4–7)	-	calib. [68]; avg. for −5.3‰ and −6.1‰ $\delta^{18}O$ seawater	not clear	[70]	
$\delta^{18}O$ bulk carbonate	ODP 765	Southern Tethys	18	52°S	~16.6 (±0.9)	~15 (±0.9)	~2 (±0.9)	calib. [68] using -1.5‰ $\delta^{18}O$ seawater	✓	[56]	
Belelemnite Mg/Ca ratio	Compilation	SE France-Spain	19	26°N	~21 (±0.6)	~19 (±0.6)	~2 (±0.6)	calib. for low Mg calcite benthic foraminifera[71]	✓	[55]	

Supplementary Table 1**. Valanginian geochemical multi-proxy based ocean temperatures across the Weissert Event.** Average values document, when possible, i) the average temperature value representing the Weissert warm interval that characterizes the initial phase of the event from the onset of the Weissert carbon isotope excursion (CIE) (A, Fig. 3 in the article) to the first Weissert CIE peak (B, Fig.3); ii) the cool temperature reached at the end of the Weissert CIE (C, Fig. 3), and iii) the temperature change (cooling) that is calculated as the difference between the ocean temperatures of both intervals. We point out that all literature records document a large scatter and thus the reported uncertainties are probably minimum estimates. This may also apply to the relatively small calibration uncertainty (e.g., 0.6/0.9) reported for oxygen isotope and Mg/Ca records.

			Summer bias assumption		Annual mean assumption	
	Time interval	N	Mean [°C]	SEM [°C]	Mean [°C]	SEM [°C]
min-TEX_{86}	Warm interval	12	21.3	1.2	24.4	1.8
	Cold end	8	18.3	1.1	21.5	1.6
	Cooling	/	3.0	1.7	2.9	2.4
max-TEX_{86}	Warm interval	12	25.5	2.7	28.7	2.8
	Cold end	8	24.4	4.2	27.6	3.9
	Cooling	/	1.1	5.0	1.0	4.7
only-TEX_{86}	Warm interval	3	21.7	0.6	23.7	2.6
	Cold end	4	20.6	0.3	24.2	1.9
	Cooling	/	1.1	0.7	-0.5	3.2
only-$\delta^{18}O$	Warm interval	4	20.7	3.1	23.6	3.5
	Cold end	4	16.1	1.6	18.7	1.9
	Cooling	/	4.7	3.5	5.0	4.0
only-Δ47	Warm interval	5	21.5	2.0	25.4	3.4
	Cold end	0	/	/	/	/
	Cooling	/	/	/	/	/

Supplementary Table 2. **Global mean surface temperature estimates (GMSTs) from model-proxy comparison of upper ocean temperatures in °C.** Mean values and standard errors of the mean (SEM) are calculated for the whole data set including the restricted basin TEX_{86} calibration for DSDP/ODP Sites 534, 603 and 692 (min-TEX_{86}), the same data but applying the BAYSPAR TEX_{86} calibration for DSDP/ODP Sites 534, 603 and 692 (max-TEX_{86}), and different subsampling experiments including only single proxy techniques (only-TEX_{86}, only-$\delta^{18}O$ and only-Δ47). The model-data comparison is performed both with annual mean model data (annual mean assumption) as well as with 3-month summer mean temperatures for sites poleward of 45 ° (summer bias assumption). The assumption of a potential bias towards summer temperatures in high-latitude proxy reconstructions always leads to lower GMST estimates due to the warmer local model temperatures (Supplementary Fig. 6).

Supplementary References

1. Gradstein, F. M. & Ogg, J. G. Chapter 2-The Chronostratigraphic Scale. in *The Geologic Time Scale* (eds Gradstein, F. M., Ogg, J. G., Schmitz, M. D. & Ogg, G. M.) 21–32 (Elsevier, Oxford 2020).

2. Ogg, J. G., Ogg, G. M., & Gradstein, F. M. *A Concise Geologic Time Scale*. (Elsevier, Amsterdam, 2016).

3. Gradstein, F., Ogg, J., Agterberg, F., Hardenbol, J. & Veen, P. van. Some Constraints on the Phanerozoic Timescale. In *Strat.* (eds. Naiwen, W. & Remane, J.) 11–19 (CRC Press, 2020).

4. Channell J.E.T., Erba E., Nakanishi M. & Tamaki K. Late Jurassic–Early Cretaceous timescales and oceanic magnetic anomaly block models. In *Geochronology, Time Scales and Global Stratigraphic Correlation* (eds Berggren W.A., Kent D.V., Aubry M.-P. and Hardenbol J.) **Vol. 54**, 51–63 (Spec. Publ. Soc. Econ. Paleontol. Mineral., 1995).

5. Malinverno, A., Hildebrandt, J., Tominaga, M., Channell, J.E.T. M-sequence geomagnetic polarity time scale (MHTC12) that steadies global spreading rates and incorporates astrochronology constraints. *J. Geophys. Res.* **117,** B6 **(**2012).

6. Sprovieri, M., Coccioni, R., Lirer, F., Pelosi, N. & Lozar, F. Orbital tuning of a lower Cretaceous composite record (Maiolica Formation, central Italy). *Paleoceanogr.* **21 (4),** PA4212 (2006).

7. Bralower, T. J., Leckie, R. M., Sliter, W. V., & Thierstein, H. R. An integrated Cretaceous microfossil biostratigraphy. In *Geochronology, Time Scales and Global Stratigraphic Correlation* (eds Berggren W.A., Kent D.V., Aubry M.-P. & Hardenbol J.) **Vol. 54**, 65–79 (Spec. Publ. Soc. Econ. Paleontol. Mineral., 1995).

8. Bown, P. R., Rutledge, D.C., Crux, J. A. & Gallagher, L. T. Early Cretaceous. In *Calcareous Nannofossil Biostratigraphy* (ed Bown, P.R.) 86–131(Chapman and Hall, Cambridge, 1998).

9. Duchamp–Alphonse, S. et al. Fertilization of the northwestern Tethys (Vocontian basin, SE France) during the Valanginian carbon isotope perturbation: evidence from calcareous nannofossils and trace element data. *Palaeogeogr. Palaeoclimatol. Palaeoecol.* **243,** 132–151 (2007).

10. Charbonnier, G. et al. Astronomical calibration of the Valanginian “Weissert” episode: The Orpierre marl–limestone succession (Vocontian Basin, southeastern France). *Cretac. Res.* **45,** 25–42 (2013).

11. Bornemann, A. & Mutterlose, J. Calcareous Nannofossil and $\delta^{13}C$ records from the Early Cretaceous of the Western Atlantic Ocean: Evidence for enhanced fertilization across the Berriasian-Valanginian transition. *Palaios* **23,** 821–832 (2008).

12. Aguado, R. et al. A new record of the Weissert episode from the Valanginian succession of Cehegín (Subbetic, SE Spain): Bio- and carbon isotope stratigraphy. *Cretac. Res.* **92,** 122–137 (2018).

13. Gréselle, B. et al. The Valanginian isotope event: A complex suite of palaeoenvironmental perturbations. *Palaeogeogr. Palaeoclimatol. Palaeoecol.* **306,** 41–57 (2011).

14. Möller, C., Mutterlose, J. & Alsen, P. Integrated stratigraphy of Lower Cretaceous sediments (Ryazanian–Hauterivian) from North-East Greenland. *Palaeogeogr. Palaeoclimatol. Palaeoecol.* **437,** 85–97 (2015).

15. Pauly, S., Mutterlose, J. & Alsen, P. Early Cretaceous palaeoceanography of the Greenland–Norwegian Seaway evidenced by calcareous nannofossils. *Mar. Micropal.* **90,** 72–85 (2012).

16. Kujau, A. et al. Reconstructing Valanginian (Early Cretaceous) mid-latitude vegetation and climate dynamics based on spore-pollen assemblages. *Rev. Palaeobot. Palynol.* **197,** 50–69 (2013).

17. Kessels, K., Mutterlose, J. & Michalzik, D. Early Cretaceous (Valanginian – Hauterivian) calcareous nannofossils and isotopes of the northern hemisphere: proxies for the understanding of Cretaceous climate. *Lethaia* **39,** 157-172 (2006).

18. Mutterlose, J. & Wise, S.W. Lower Cretaceous nannofossil biostratigraphy of ODP Leg 113 Holes 692B and 693A, continental slope off East Antarctica, Weddell Sea. In *Proc. ODP Sci. Res.* **Vol. 113** (eds Barker, P. R, Kennett, J. P., et al.,) 325–351 (Ocean Drilling Program, 1990).

19. Roth, P.H. Jurassic and Lower Cretaceous calcareous nannofossils in the western North Atlantic (Site 534): biostratigraphy, preservation, and some observations on biogeography and

palaeoceanography. In *Init. Repts. DSDP* **Vol. 76** (eds Sheridan, R.E. & Gradstein, F.M.) 587–621 (Deep Sea Drilling Project, 1983).

20. Channell, J. E. T., Erba, E., & Lini, A. Magnetostratigraphic calibration of the Late Valanginian carbon isotope event in pelagic limestones from Northern Italy and Switzerland. *Earth and Planet. Sci. Lett.* **118 (1–4),** 145–166 (1993).

21. Erba, E., Bartolini, A. & Larson, R. L. Valanginian Weissert oceanic anoxic event. *Geology* **32,** 149 (2004).

22. Barbarin, N. et al. Evidence for a complex Valanginian nannoconid decline in the Vocontian basin (South East France). *Mar. Micropaleontol.* **84,** 37–53 (2012).

23. Jeremiah, J. A Lower Cretaceous nannofossil zonation for the North Sea Basin. *J. Micropal.* **20,** 45–80 (2001).

24. Thierstein, H. R. Mesozoic calcareous nannoplankton biostratigraphy of marine sediments. *Mar. Micropal.* **1,** 325–362 (1976).

25. Erba, E. & Quadrio, B. Biostratigrafia a Nannofossili calcarei, Calpionellidi e Foraminiferi planctonici della Maiolica (Titoniano superiore-Aptiano) nelle Prealpi Bresciane (Italia Settentrionale). *Rivista Italiana Paleontologia Stratigr*afia **93,** 3–108 (1987).

26. Littler, K., Robinson, S. A., Bown, P. R., Nederbragt, A. J. & Pancost, R. D. High sea-surface temperatures during the Early Cretaceous Epoch. *Nat. Geosci.* **4,** 169–172 (2011).

27. Applegate, J. L., & Bergen, J. A. Cretaceous calcareous nannofossil biostratigraphy of sediments recovered from the Galicia Margin, ODP Leg 103. In *Proc. ODP Sci. Res.* **Vol. 103** (eds Boillot, G., Winterer, E. L., et al.) 293–348 (Ocean Drilling Project, 1988).

28. Mutterlose, J. Lower Cretaceous nannofossil biostratigraphy off northwestern Australia (Leg 123). In *Proc. ODP, Sci. Res.* **Vol. 123** (eds Gradstein, F. M., Ludden, J. N., et al.) 343–368 (Ocean Drilling Project, 1992).

29. Bown, P. R. New calcareous nannofossil taxa from the Jurassic/Cretaceous boundary interval of Sites 765 and 261, Argo Abyssal Plain. In *Proc. ODP Sci. Res.* **Vol. 123** (eds Gradstein, F. M., Ludden, J. N., et al.) 369–380 (Ocean Drilling Project, 1992).

30. Bersezio, R., Erba, E., Gorza, M. & Riva, A. Berriasian–Aptian black shales of the Maiolica formation (Lombardian Basin, Southern Alps, Northern Italy): local to global events. *Palaeogeogr. Palaeoclimatol. Palaeoecol.* **180,** 253–275 (2002).

31. Erba, E. & Tremolada, F. Nannofossil carbonate fluxes during the Early Cretaceous: Phytoplankton response to nutrification episodes, atmospheric CO_2, and anoxia. *Paleoceanogr.* **19 (1),** PA1008 (2004).

32. Channell, J. E. T., Erba, E., Muttoni, G. & Tremolada, F. Early Cretaceous magnetic stratigraphy in the APTICORE drill core and adjacent outcrop at Cismon (Southern Alps, Italy), and correlation to the proposed Barremian–Aptian boundary stratotype. *Geol. Soc. Am. Bull.* **112 (9),** 1430–1443 (2000).

33. Duchamp-Alphonse, S., Gardin, S. & Bartolini, A. Calcareous nannofossil response to the Weissert episode (Early Cretaceous): Implications for palaeoecological and palaeoceanographic reconstructions. *Mar. Micropaleontol.* **113,** 65–78 (2014).

34. Barbu, V. & Melinte-Dobrinescu, M. C. Latest Jurassic to earliest Cretaceous paleoenvironmental changes in the Southern Carpathians, Romania: regional record of the late Valanginian nutrification event. *Cretac. Res.* **29,** 790–802 (2008).

35. Covington, J. M., & Wise, S. W. Calcareous nannofossil biostratigraphy of a Lower Cretaceous deep-sea fan complex: Deep Sea Drilling Project Leg 93 Site 603, lower continental rise off Cape Hatteras. In *Init. Repts. DSDP* **Vol. 93** (eds van Hinte, J. E., Wise, S. W., Jr., et al.) 617–660 (Deep Sea Drilling Project, 1987).

36. Gran, H. H., & Braarud, T. A quantitative study of the phytoplankton in the Bay of Fundy and the Gulf of Maine (including observations on hydrography, chemistry and turbidity). *J. Biol. Board Canada* **1 (5),** 279–467 (1935).

37. Smayada, T. J. A quantitative analysis of the ecological conditions and the phytoplankton dynamics at 8° 45PN, 79° 23PW from November 1954 to May 1957. *Inter-American Tropical Tuna Commission Bulletin* **2,** 353–612 (1966).

38. Bukry, D. Coccoliths as paleosalinity indicators–Evidence from Black Sea. In *The Black Sea–Geology, Chemistry, and Biology* (eds. Degens E.T. & Ross D.A.) **Vol. 20**, 353–363 (Am. Assoc. Petrol. Geol., Mem., 1974).

39. Peleo–Alampay, A. M., Mead, G. A., & Wei, W. Unusual Oligocene Braarudosphaera-rich layers of the South Atlantic and their palaeoceanographic implications. *J. Nannoplankton Res.* **21 (1),** 17–26 (1999).

40. Scholle, P. A., & Wenkam, C. R. Geological studies of the COST Nos. G-1 and G-2 wells, United States North Atlantic Outer Continental Shelf. *US. Geol. Soc.* Circular 861 (1982).

41. Riegraf, W. Benthonische Schelf-Foraminiferen aus dem Valanginium-Hauterivium (Unterkreide) des Indischen Ozeans südwestlich Madagaskar (Deep Sea Drilling Project Leg 25, Site 249). *Geologische Rundschau* **78 (3),** 1047–1061 (1989).

42. Koutsoukos, A.M. Mid to Late Cretaceous microbiostratigraphy, palaeoecology and palaeogeography of the Sergipe basin, Northeastern Brazil. *Ph.D. thesis, University of Rio de Janeiro* (1989).

43. Frenzel, P. Die benthischen Foraminiferen der Rügener Schreibkreide (Unter-Maastricht, NE-Deutschland). *Neue Paläontologische Abhandlungen* **3,** 1–361 (2000).

44. Holbourn, A. E. L. & Kaminski, M. A. Valanginian to Barremian Benthic Foraminifera from ODP Site 766 (Leg 123, Indian Ocean). *Micropal.* **41,** 197 (1995).

45. Van Morkhoven, F. P., Berggren, W. A., Edwards, A. S., Oertli, H. J. Cenozoic cosmopolitan deep-water benthic foraminifera. *Bull. Centres Rech. Explor.–Prod. Elf-Aquitaine, Mem.* **11,** 68–70 (1986).

46. Gillmore, G.K., Kjennerud, T. and Kyrkjebo, R. The reconstruction and analysis of palaeowater depths: a new approach and test of micropalaeontological approaches in the post-rift (Cretaceous to

Quaternary) interval of the northern North Sea. In *Sedimentary Environments Offshore Norway - Palaeozoic to Recent* (eds Martinsen, O.J. & Dreyer, T.) No.10, 365–381 (Norw. Petr. Soc. Spec. Pub., 2001).

47. O'Brien, C. L. et al. Cretaceous sea-surface temperature evolution: Constraints from TEX86 and planktonic foraminiferal oxygen isotopes. *Earth Sci. Rev.* **172,** 224–247 (2017).

48. Trommer, G. et al. Distribution of Crenarchaeota tetraether membrane lipids in surface sediments from the Red Sea. *Org. Geochem.* **40,** 724–731 (2009).

49. Kim, J.–H. et al. Influence of deep-water derived isoprenoid tetraether lipids on the TEX_{86}^{H} paleothermometer in the Mediterranean Sea. *Geochim. Cosmochim. Ac.* **150,** 125–141 (2015).

50. Tierney, J. E. & Tingley, M. P. A TEX_{86} surface sediment database and extended Bayesian calibration. *Sci. Data* **2**, 150029 (2015).

51. Steinig, S. et al. Evidence for a regional warm bias in the Early Cretaceous TEX86 record. *Earth Planet. Sci. Lett.* **539,** 116184 (2020).

52. Polik, C. A., Felix J. E. & Ann Pearson. Impacts of Paleoecology on the TEX_{86} Sea Surface Temperature Proxy in the Pliocene-Pleistocene Mediterranean Sea. *Paleoceanogr. And Palaeoclimatol*. **33 (12)**, 1472–1489 (2018).

53. Schouten, S., Hopmans, E. C. & Sinninghe Damsté, J. S. The effect of maturity and depositional redox conditions on archaeal tetraether lipid palaeothermometry. *Org. Geochem.* **35,** 567–571 (2004).

54. Farnsworth, A. et al. Climate Sensitivity on Geological Timescales Controlled by Nonlinear Feedbacks and Ocean Circulation. *Geophys. Res. Lett.* **46,** 9880–9889 (2019).

55. Price, G. D. et al. A High-Resolution Belemnite Geochemical Analysis of Early Cretaceous (Valanginian–Hauterivian) Environmental and Climatic Perturbations. *Geochem. Geophys. Geosyst.* **19,** 3832–3843 (2018).

56. Charbonnier, G. et al. A global palaeoclimatic reconstruction for the Valanginian based on clay mineralogical and geochemical data. *Earth-Sci. Rev.* **202,** 103092 (2020).

57. Bodin, S., Meissner, P., Janssen, N. M. M., Steuber, T. & Mutterlose, J. Large igneous provinces and organic carbon burial: Controls on global temperature and continental weathering during the Early Cretaceous. *Glob. Planet. Change* **133,** 238–253 (2015).

58. Meissner, P., Mutterlose, J. & Bodin, S. Latitudinal temperature trends in the northern hemisphere during the Early Cretaceous (Valanginian–Hauterivian). *Palaeogeogr. Palaeoclimatol. Palaeoecol.* **424,** 17–39 (2015).

59. Tierney, J. E. & Tingley, M. P. A Bayesian, spatially-varying calibration model for the TEX_{86} proxy. *Geochim. Cosmochim. Ac.* **127**, 83–106 (2014).

60. Foster, G. L., Royer, D. L. & Lunt, D. J. Future climate forcing potentially without precedent in the last 420 million years. *Nat. Commun.* **8,** 14845 (2017).

61. Thiede, D. S. & Vasconcelos, P. M. Paraná flood basalts: Rapid extrusion hypothesis confirmed by new 40Ar/39Ar results. *Geology* **38,** 747–750 (2010).

62. Janasi, V. de A., de Freitas, V. A. & Heaman, L. H. The onset of flood basalt volcanism, Northern Paraná Basin, Brazil: A precise U–Pb baddeleyite/zircon age for a Chapecó-type dacite. *Earth Planet. Sci. Lett.* **302,** 147–153 (2011).

63. Dodd, S. C., Mac Niocaill, C. & Muxworthy, A. R. Long duration (>4 Ma) and steady-state volcanic activity in the early Cretaceous Paraná–Etendeka Large Igneous Province: New palaeomagnetic data from Namibia. *Earth Planet. Sci. Lett.* **414,** 16–29 (2015).

64. Rayner, N. A. Global analyses of sea surface temperature, sea ice, and night marine air temperature since the late nineteenth century. *J. Geophys. Res.* **108,** 4407 (2003).

65. Petersen, S.V. et al. Effects of improved ^{17}O correction on interlaboratory agreement in clumped isotope calibrations, estimates of mineral-specific offsets, and temperature dependence of acid digestion fractionation. *Geochem. Geophys. Geosyst.* **20,** 3495–3519 (2019).

66. Price, G. D., Bajnai, D. & Fiebig, J. Carbonate clumped isotope evidence for latitudinal seawater temperature gradients and the oxygen isotope composition of Early Cretaceous seas. *Palaeogeogr. Palaeoclimatol. Palaeoecol.* **552,** 109777 (2020).

67. Price, G. D. & Passey, B. H. Dynamic polar climates in a greenhouse world: Evidence from clumped isotope thermometry of Early Cretaceous belemnites. *Geology* **41**, 923–926 (2013).

68. Anderson, T.F. & Arthur, M.A. Stable isotopes of oxygen and carbon and their application to sedimentologic and paleoenvironmental problems. In *Stable Isotopes in Sedimentary Geology* (eds Arthur, M.A., Anderson, T.F., Kaplan, I.R., Veizer, J. & Land, L.S.) 1–151 (SEPM Short Course, Tulsa, 1983).

69. Gómez Dacal, A. R. et al. Evidence of warm seas in high latitudes of southern South America during the Early Cretaceous. *Cretac. Res.* **95,** 8–20 (2019).

70. Price, G. D. & Nunn, E. V. Valanginian isotope variation in glendonites and belemnites from Arctic Svalbard: Transient glacial temperatures during the Cretaceous greenhouse. *Geology* **38,** 251–254 (2010).

71. Lear, C. H., Rosenthal, Y. & Slowey, N. Benthic foraminiferal Mg/Ca-paleothermometry: A revised core-top calibration. *Geochim. Cosmochim. Ac.* **66,** 3375–3387 (2002).
